# A pseudorabies outbreak in hunting dogs in Campania region (Italy): a case presentation and epidemiological survey

**DOI:** 10.1186/s12917-024-04189-3

**Published:** 2024-07-18

**Authors:** Gianmarco Ferrara, Ugo Pagnini, Antonio Parisi, Maria Grazia Amoroso, Giovanna Fusco, Giuseppe Iovane, Serena Montagnaro

**Affiliations:** 1 Department of Veterinary Medicine and Animal Productions, University of Naples Federico II, Via Federico Delpino n.1, Naples, 80137 Italy; 2 Department of Animal Health—Istituto Zooprofilattico Sperimentale del Mezzogiorno, Via Salute n. 2, Naples, 80055 Italy

**Keywords:** Pseudorabies virus, Hunting dogs, Suid herpesvirus, Wild boar, Aujeszky’s disease

## Abstract

**Background:**

Pseudorabies is an infection of domestic and wild pigs that has occasionally been reported in dogs with fatal encephalitis. Hunting dogs are predisposed to pseudorabies exposure due to incorrect practices (administration of raw infected meat) or close contact with infected wild boars. This study described an outbreak of pseudorabies in two hunting dogs in the Campania region, southern Italy.

**Case presentation:**

Two hunting dogs were hospitalized after a hunting trip, with fever, itching, and self-inflicted lesions. Laboratory tests showed mild anemia and marked leukocytosis. Despite conservative therapy, both animals died 48 h after the presentation of symptoms. One of the carcasses was sent to the Department of Veterinary Medicine and Animal Production in Naples to confirm the suspicion of pseudorabies. DNA was extracted from different matrices and used as a template for real-time PCR to detect PRV. Several samples (brain, cerebellum, brainstem, lung, and liver) tested positive. Subsequent sequence analyses of glycoprotein E from DNA extracted from the brain stem revealed a sequence similarity to those described in previous cases of pseudorabies in dogs in Italy, France and Belgium. One month after the outbreak, blood samples were collected from 42 dogs belonging to the same hunting team and from 245 dogs (cohort population) living in the Campania region. All samples were tested with two commercial ELISAs to detect seroconversion against glycoproteins B and E. A seroprevalence of 19% was observed in the hunting team affected by the outbreak, while only 0.8% was observed in the regional dog population.

**Conclusions:**

The data reported in this study demonstrate potential exposure to PRV by dead-end hosts, particularly hunting dogs. The sequencing results indicated the homogeneity of PRV strains circulating in the different Italian regions.

**Supplementary Information:**

The online version contains supplementary material available at 10.1186/s12917-024-04189-3.

## Background

Pseudorabies, also called Aujeszky’s disease (AD), is an infection caused by Varicellovirus suidalpha1 (SuHV-1 or PRV), an enveloped DNA virus belonging to the family *Orthoherpesviridae*, subfamily *Alphaherpesvirinae*, genus *Varicellovirus* [1]. Since domestic and wild swine are susceptible to productive infection, they are considered the natural hosts and reservoirs of PRV that can transmit the virus to other mammals (namely dead-end hosts) [1]. The disease is characterized by mild or severe respiratory symptoms in adult pigs and reproductive failure in pregnant sows, while neurological syndromes have also been described (due to encephalomyelitis) in piglets or predisposed animals. In this species, PRV can determine a latency period by entering the neurological system (in the nerve ganglia) and remaining there until reactivation (occurring after stress or other external causes), as reported in other Herpesviridae members [2–4].

AD is recognized worldwide as a cause of economic losses, and several countries have conducted extensive vaccination campaigns for its eradication in domestic pigs (including Italy, where PRV has been notifiable since 1997) [5, 6]. Wild boars (*Sus scrofa*) play a key role in the persistence of this infection in Europe (serological and molecular evidence has been described), especially in the last decades due to the emergence of new and less pathogenic strains [1, 7]. Furthermore, the increasing number of wild boars in Europe and the overlap of domestic and wild animal habitats further facilitate the risk of transmission [8].

AD has not yet completely eradicated in the Campania region [6]. Additionally, PRV has been demonstrated to be highly prevalent in wild boar in the Campania region, representing an important issue for dead-end hosts [6, 9].

Dead-end hosts, among which carnivores are particularly susceptible, become infected by exposure to infected wild boars [10]. Dogs (especially hunting dogs), wolves, and foxes commonly acquire the infection by the digestive route (through bites or feeding infected raw meat or viscera), even if the respiratory route is also described [8, 11–13]. PRV causes severe and highly lethal encephalomyelitis in dead-end hosts due to its high neurotropism. Death usually occurs within 1–2 days and follows a characteristic pruritus of the head, known as “mad itch” [10, 14]. A clinical history consistent with direct or indirect contact with pigs or wild boars, as well as the appearance of neurological symptoms associated with itching, may raise a suspicion of PRV in dogs [15]. Etiological confirmation is performed only post-mortem using virus isolation, polymerase chain reaction (PCR) on the nervous system or other organs [16]. Clinical occurrences of PRV in dogs have been recorded all throughout the world, particularly in regions where SuHV-1 circulates among domestic pigs and wild boars [5, 14–17]. In this case report, we described the occurrence of AD in two hunting dogs in the Campania region (southern Italy) after contact with wild boars, reporting the progression of the infection and laboratory findings. Furthermore, the exposure of other dogs belonging to the same hunting team to PRV was also described and compared to that of the cohort population.

## Case presentation

**Fig. 1:**
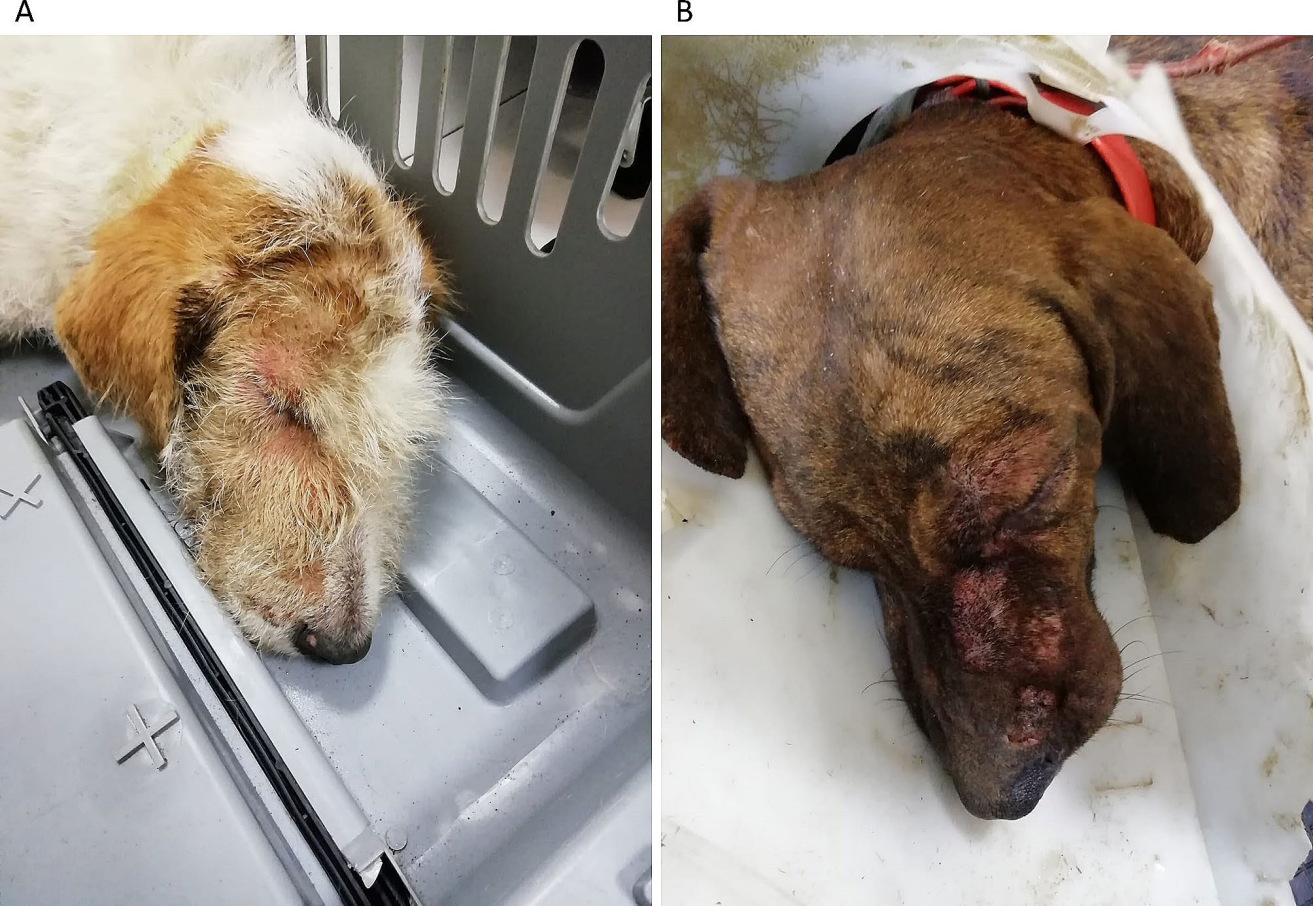
Legend: Self-made injuries due to intense itching in Pupa **(A)** and Tigre **(B).** Both animals had abrasions in the periorbital and labial regions

**Fig. 2:**
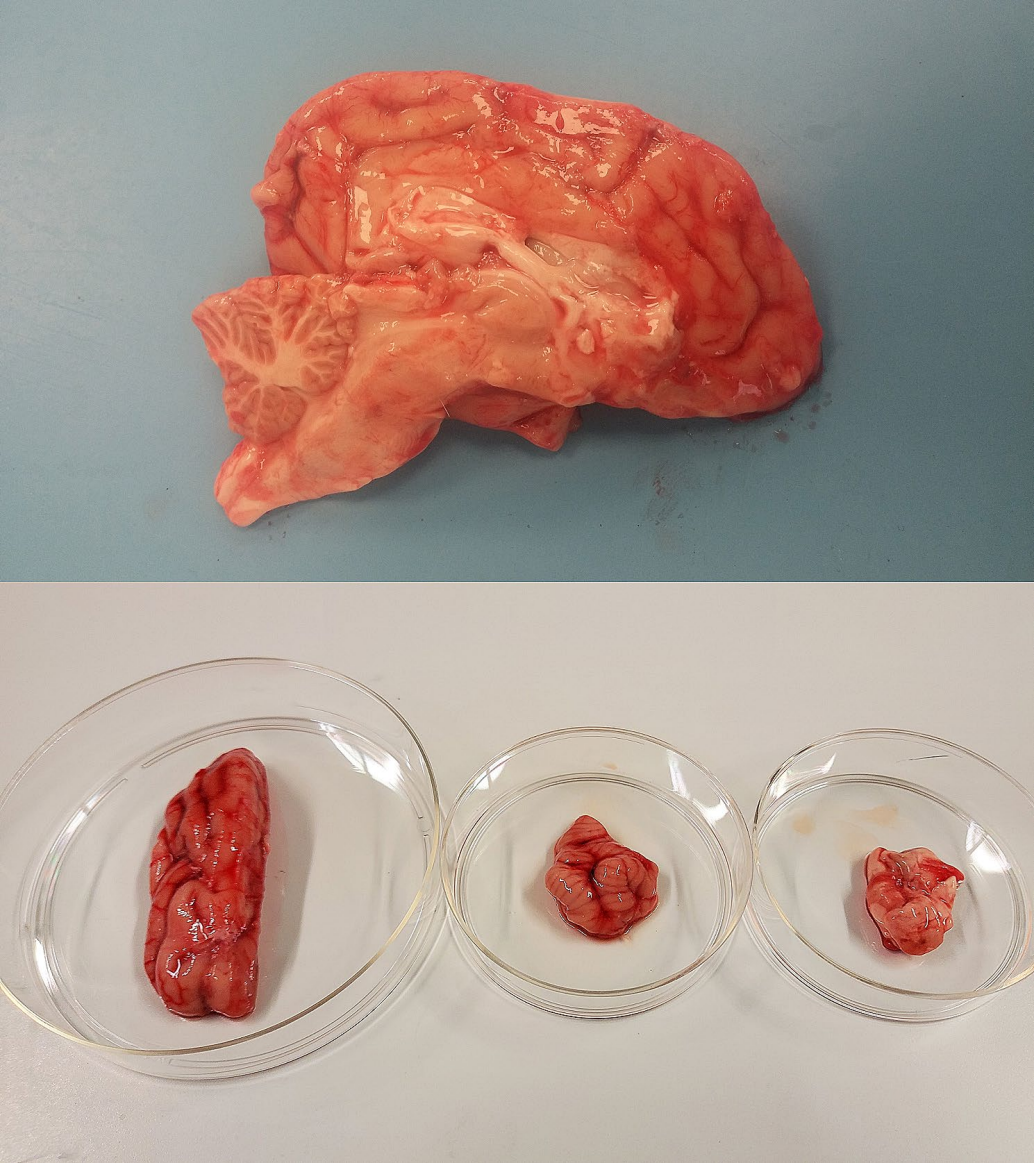
Legend: Appearance of the brain (left), cerebellum (center) and brainstem (right) of Pupa after death due to PRV infection. Evidence of hyperemia affecting the meninges can be noted

**Fig. 3:**
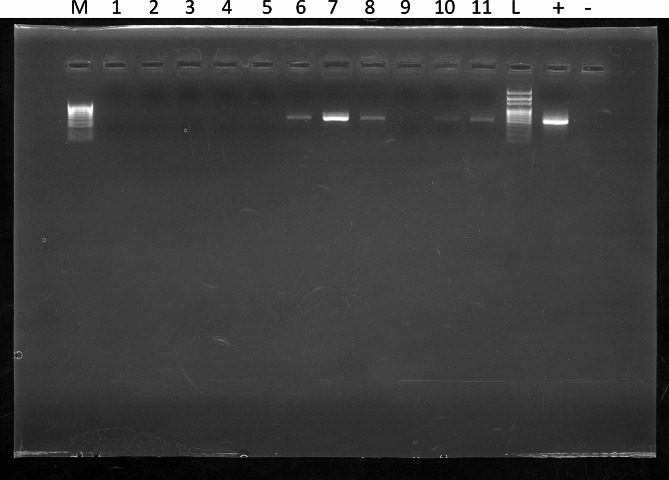
Legend: Electrophoresis of PCR amplicons targeting gE partial sequence using a 1.2% agarose gel. M = molecular marker (1000 –100 bp), 1 = blood, 2 = serum; 3 = feces, 4 = gastric contents, 5 = tonsils, 6 = brain, 7 = brain stem, 8 = cerebellum, 9 = spleen, 10 = lungs, 11 = liver, L = ladder (3000 –100 bp), + = positive control, − = negative control. A band of approximately 500 bp is observable in brain-derived samples (6, 7, and 8), while it is weaker in the lungs and liver. The other samples tested negative

**Fig. 4:**
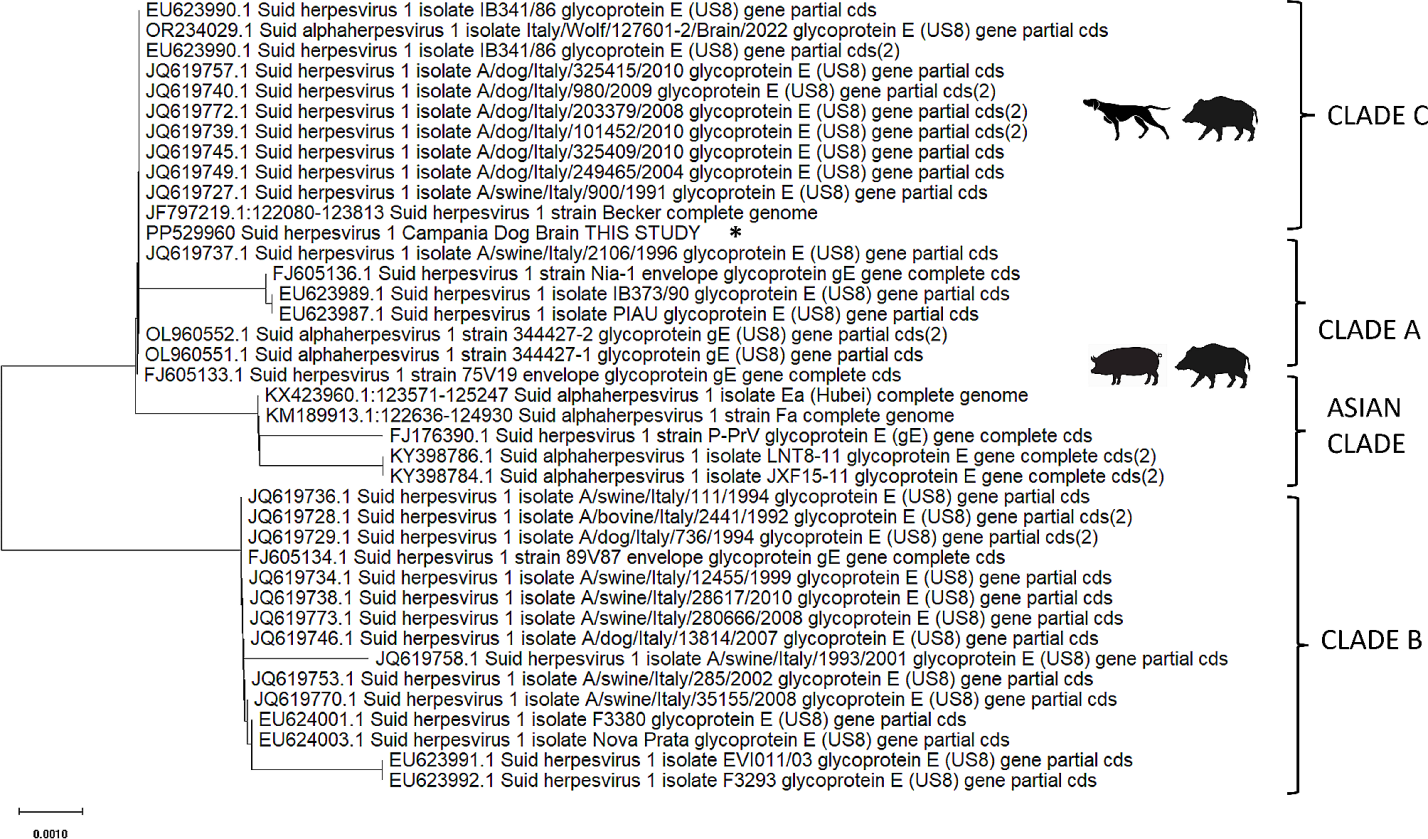
Legend: Phylogenetic tree based on partial sequencing of the US8 gene (glycoprotein E sequence). The tree was obtained using the maximum likelihood method in MEGA 10 software. The analysis revealed the homology of the strains of PRV identified in different outbreaks in carnivores in Italy. The sequence found in the present study was indicated with an asterisk. The proposed phylogeny was taken by previous studies [12, 20]

This case report was described in Campania (40◦49′34″N 14◦15′23″E), a region where pig farming was practiced and sporadic evidence of PRV infections was described. Furthermore, it is one of the Italian regions with the biggest number of wild boars and dogs, consequently, many dogs have been trained specifically for wild boar hunting. Two Istrian short-haired hounds (Pupa, a 5-year-old female, and Tigre, a 2-year-old male) from Salerno, a province of Campania (southern Italy), were hospitalized in a private veterinary hospital on December 6, 2019 for fever, neurological signs, incoercible itch, and severe self-made facial lesions. According to the anamnesis, the symptoms began a few days after a hunting trip in the same Italian region. Clinical examination revealed the presence of fever, ataxia, generalized muscular tremors, and self-inflicted erosions of the skin in the region of the head in both animals (Fig. 1 and Additional file 1). Two aliquots of blood (one with anticoagulant and one without) belonging to both animals were centrifuged and stored at -20 °C for future molecular analysis. Complete blood count and blood chemistry (performed using a Procyte Dx Haematology Analyser, IDEXX, US) were comparable between the two animals and showed regenerative and hypochromic anemia, reduced hematocrit, and a marked leukocytosis characterized by neutrophilia and a mild monocytosis (Table 1). Despite the conservative therapies (based on fluid therapy and butorphanol), the clinical presentation did not change, and both animals died 48 h from the start of clinical manifestations. Based on the anamnesis and clinical signs of the animals, pseudorabies was suspected as the cause of the death of both animals. One carcass (Pupa, a 5-year-old female) was sent to the Department of Veterinary Medicine and Animal Production of Naples (Naples, Italy) for a definitive etiological diagnosis. Except for the presence of facial lesions, the dead dog was in good nutritional condition and showed no noteworthy abnormalities. The thoracic and abdominal cavities, as well as the viscera, were examined and found to be normal. Examination of the brain, cerebellum, and encephalic trunk revealed hyperemia and congestion (Fig. 2). DNA was extracted from several specimens, such as blood, serum, feces, gastric contents, tonsils, brain, brainstem, cerebellum, spleen, lungs, and liver, using DNeasy Blood & Tissue Kits QIAGEN (Qiagen, Germany) following the manufacturer’s instructions. The reliability of each purified and quantified (NanoDrop 2000, Thermo Scientific, US) sample was assessed using end-point PCR with GAPDH as the housekeeping gene. Each DNA was used as template in a real-time PCR reaction consisting of a 20-µL reaction volume including 10 µL of 2X SYBR Green (BioRad, US), 20 µM of each primer (forward: 5′-TCTCGGACATGGGCGACT-3′; reverse: 5′-CACGTAGTACAGCAGGCAC-3′), 50 ng DNA, and ddH2O to final volume [18]. Amplification conditions included initial denaturation at 95 °C for 5 min, 40 cycles of 95 °C denaturation for 15 s, 56 °C annealing for 15 s, and 72 °C extension for 15 s. This protocol amplified a conserved region of the gE genes of PRV (92 base pairs) [18]. DNA extracted from PRV Suid herpesvirus 1 strain VR-1362 (ATCC) was used as a positive control and the PCR mix without template DNA represented the negative control. Rabies (however absent in Italy) infection was excluded using an immunofluorescent protocol described in the literature [19]. Table 2 summarizes the positivity and cycle threshold (Ct) values of the different organs and tissues and identified the brainstem as the sample with the lowest Ct value. All samples were further tested with a PCR targeting the partial sequence of gE (493-bp) and commonly used for phylogenetic analysis [19]. Cycling conditions (T100 Thermal Cycler, BioRad, US) and primers were as follows: denaturation at 95 °C for 5 min, 45 cycles of 95 °C for 50 s, 60 °C for 40 s, and 72 °C for 50 s, and final extension at 72 °C for 5 min, forward primer 5′-CCGCGGGCCGTGTTCTTTGT-3′, and reverse primer 5′-CGTGGCCGTTGTGGGTCAT-3′ [19]. The amplified products were run in a 1.2% agarose gel (Certified Molecular Biology Agarose, BioRad, US) and visualized with a UV reader. The brain stem was also the most positive sample in this PCR, therefore the amplification product was purified using a commercial kit (QIAquick PCR Purification Kit, Qiagen, Germany) and sequenced (by an external service, BMR Genomics, Italy) using the Sanger method (Fig. 3). Sequence data were analyzed using BLAST to identify similarities with GenBank sequences. Sequencing revealed 100% homology with PRV strains causing outbreaks in France, Belgium and Sicily (clade c) [5, 17, 20, 21]. Phylogenetic analysis was conducted using the maximum likelihood (ML) method in molecular evolutionary genetics software version 10 (MEGA 10) (Fig. 4).

**Table 1:** Laboratory findings from two dogs infected with PRV (complete blood count and hematobiochemical test)

Parameter	Pupa	Tigre
Erythrocytes	**4.64** (M/µL)	**5.44** (M/µL)
Hematocrit	**27.7** (%)	**34.3** (%)
Hemoglobin	**10.2** (g/dL)	**12.7** (g/dL)
MCV	**59.7** (fL)	63.1 (fL)
MCH	22 (pg)	23.3 (pg)
MCHC	36.8 (g/dL)	37 (g/dL)
RDW	17 (%)	15.7 (%)
Reticulocytes	0.9 (%)	0.6 (%)
Leukocytes	**89.2** (K/µL)	**39.55** (K/µL)
Neutrophiles	**77.41** (K/µL)	**34.87** (K/µL)
Limphocytes	4.56 (K/µL)	2.69 (K/µL)
Monocytes	**7.06** (K/µL)	**1.86** (K/µL)
Eosinophils	0.03 (K/µL)	0.07 (K/µL)
Basophils	0.16 (K/µL)	0.06 (K/µL)
Platelets	**624** (K/µL)	278 (K/µL)
Glycemia Creatinine	56 (mg/dL) 0.6 (mg/dL)	/ /
Urea	14 (mg/dL)	/
Na	152 (mmol/L)	/
K	**3.4** (mmol/L)	/
Total protein	7.2 (g/dL)	/
Albumin	3.1 (g/dL)	/
Globulin	4.1 (g/dL)	/
ALT	58 (U/L)	/
ALP	142 (U/L)	/

**Table 2:** Results obtained testing different specimens in the SYBR real-time PCR. POS = positive sample, NEG = negative sample, ct = cycle threshold

Specimes	gE outcome	Ct
Blood	NEG	/
Serum	NEG	/
Feces	NEG	/
Gastric contents	NEG	/
Tonsils	NEG	/
Brain	POS	28.11
Brain stem	POS	20.38
Cerebellum	POS	27.91
Spleen	NEG	/
Lungs	POS	33.16
Liver	POS	29.21

One month following the outbreak, blood samples were collected from all the dogs of the involved hunting team (*n* = 42) and from a representative sample of the dog population in Campania (*n* = 245, including pets, stray and hunting dogs). The sample size of 287 dogs was calculated using the formula proposed by Thurshfield for a theoretically “infinite” population with the following specifications: expected prevalence of PRV 5%, 95% confidence interval (CI) and desired absolute precision (5%). Each sample was centrifuged (1000 g x 15 min), and the serum recovered was analyzed for the presence of antibodies to PRV gB and gE using two commercial and competitive ELISAs (IDEXX PRV/ADV gB Ab and IDEXX PRV/ADV gE Ab; IDEXX, US) which have been validated in pigs and successfully used in dogs and other carnivores [11, 22]. Both tests, which represent a quick and effective tool to highlight PRV exposure, were performed following the manufacturer’s instructions and reading the optical density (450 nm) using a spectrophotometer (Multiskan™ FC Microplate Photometer, Thermo-Scientific, US). A total of eight dogs (19%) tested positive for the identification of gE and gB antibodies (Table 3). The same tests were also subjected to a representative sample of the Campania canine population in which only two animals (0.8%) tested positive (both strays) (Table 3) [23]. The hunting team’s animals showed a significantly higher prevalence than the rest of the analyzed sample, indicating that these dogs were exposed to PRV (Table 3).

**Table 3:** PRV seroprevalence in animals belonging to the hunting team involved in the outbreak and cohort population

	Total (*n*)	gE Positive (*n*)	gB Positive (*n*)	Prevalence (%)	CI 95%	Fisher	*p*
Hunting team	42	8	8	19	7.2–30.9	0.35	< 0.001
Cohort	245	2	2	0.8	0–1.9		

## Discussion and conclusions

In this study, we described the occurrence of pseudorabies in two hunting dogs in the Campania region of southern Italy. Because the carcass of one dog was immediately sent to the “Department of Veterinary Medicine and Animal Production” laboratories (Naples, Italy), the cases presented in this study provide an uncommon opportunity for determining a diagnosis and represent the first well-documented AD reports in hunting dogs in Campania. In the same region, PRV was already identified as the cause of death of a wolf in 2018 [24]. Furthermore, the infection has already been reported in recent years in other regions of southern Italy (such as Sicily) [13, 20, 25]. Our study shared with these recent outbreaks both the symptoms (similar to that observed in previous cases of AD in dogs) and the PRV strain involved (a homology of 100% was found) [20]. The high degree of similarity with isolates reported in previous investigations in Italy was expected given that PRV is stable in the regions in which it circulates.

In other cases of interspecific transmission in dogs, the animals died within 48 h of exposure. Our work provided further information on the laboratory findings (blood count and blood chemistry test results) as well as documented the symptoms via multimedia. Although the origin of this outbreak was unknown, close contact between infected wild boars and hunting dogs or the consumption of contaminated raw meat and viscera (however excluded by the anamnesis) could be hypothesized [8]. Experiments on the development of AD in dogs and other non-natural hosts reveal that the different location of pruritus corresponds to the viral route of entry. When infection occurs through the oral and/or respiratory mucosa, the head and neck are most implicated [10]. We can therefore hypothesize that transmission took place via these routes. Recent studies have also highlighted through animal experiments that dogs can transmit the infection to other conspecifics (including the vaccine strains) in particular conditions [26]. In the absence of information about the real source of infections, it was possible to hypothesize that the two infected animals came into contact with the same source [10]. The other hypothesis included that only the first dog (Pupa) came into contact with the source of infection and then transmitted the virus to the second dog. The detection of antibodies against gB and gE in 19% of the animals belonging to the same hunting group (significantly higher than the 0.9% of positive animals in the dog population of the same region) provided further support for this hypothesis. Unfortunately, we were unable to determine whether the seroconversion was caused by the same outbreak or previous interactions with the virus to which the animal was exposed during its lifetime because we did not have a duplicate serum sample (just one taken 30 days after the epidemic) [22]. Despite the limitations of this study, which included the inability to conduct additional molecular analyses of the PRV strains as well as the lack of wide sampling in hunting teams used for serosuvery, this case report described several aspects of a PRV outbreak in dogs.

Wild animals represent a reservoir of innumerable infections and constitute a health threat for humans and domestic animals [27–29]. Pseudorabies is one of the infections transmissible from wildlife to domestic animals, and cases of this infection in dogs are regularly reported in countries where PRV is endemic in domestic or feral pigs [1, 5]. Campania has a large wild boar population and hunting is considered a common practice. Previous studies revealed a seroprevalence of 23.85% and a molecular prevalence of 1.6% in reproductive tissues among hunted wild boars, highlighting the risk of infection for hunting dogs from this region [6, 9]. The infection is also still present in the population of domestic pigs on Italian territory. All these conditions result in occasional transmission of the infection in dogs, with hunting dogs being predisposed for epidemiological reasons. In recent years, there have been numerous reports of this infection in dogs, in particular in Sicily, other cases of PRV were described in hunting dogs due to the ingestion of raw wild boar meat [13]. As a result, significant emphasis should be focused on managing AD in natural reservoir hosts, and hunters should be more careful to avoid close contact between their animals and prey, as well as to avoid feeding their animals meat and offal from hunting.

### Electronic supplementary material

Below is the link to the electronic supplementary material.

Supplementary Material 1. **Additional file 1**: Clinical presentations of two hunting dogs infected with PRV.

## Data Availability

Sequence data that support the findings of this study have been deposited in the GenBank (NCBI, National Library of Medicine, U.S.) with the primary accession code (PP529960).

## Abbreviations

AD: Aujesky disease
SuHV-1: Suid herpesvirus-1
PRV: Pseudorabies virus
DNA: Deoxyiribonucleic acid
PCR: Polymerase chain reaction
ELISA: Enzyme linked immunosorbent assay
